# Comment on: On the role of data, statistics and decisions in a pandemic statistics for climate protection and health—dare (more) progress!

**DOI:** 10.1007/s10182-022-00447-7

**Published:** 2022-05-21

**Authors:** Walter J. Radermacher

**Affiliations:** Ludwig-Maximilians-Universitat München Fakultät für Mathematik Informatik und Statistik, Munich, Germany

## Abstract

In the Corona pandemic, it became clear with burning clarity how much good quality statistics are needed, and at the same time how unsuccessful we are at providing such statistics despite the existing technical and methodological possibilities and diverse data sources. It is therefore more than overdue to get to the bottom of the causes of these issues and to learn from the findings. This defines a high aspiration, namely that firstly a diagnosis is carried out in which the causes of the deficiencies with their interactions are identified as broadly as possible. Secondly, such a broad diagnosis should result in a therapy that includes a coherent strategy that can be generalised, i.e. that goes beyond the Corona pandemic.

## How statistics can help

Given the urgency of the Corona pandemic, it seems natural to focus the question of the role of statistics on this. In the article “*On the role of data, statistics and decisions in a pandemic*” (Jahn et al. 2022), a cooperation of authors from very different fields pursues the very commendable approach of compiling the essential findings for statistics in a kind of inventory after about two years of the pandemic. In this short contribution, however, a broader perspective and a longer horizon are advocated (Radermacher 2020, 2021a). It is above all the need for transformation towards sustainable development in many areas of life, in production and consumption, in mobility and nutrition, etc., that will call for new statistics as a basis for political decisions and discourse in the years to come. We must adapt to this as quickly as possible so that the quality of the facts meets the demands (in time). In a strategic approach that aims to help statistics achieve its potential and adequate validity in its role for modern societies in coping with their problems, it is crucial that the different facets of this discipline are meaningfully linked. It is by no means sufficient to string together method development, applications, etc. as isolated elements. Rather, it is important to combine these puzzle elements into an overall picture of a coordinated and balanced strategy contained therein.^1^ The “Data Manifesto” (Royal Statistical Society 2019) of the Royal Statistical Society shows that it is possible to bring together the various sectors of the statistical community with their specific perspectives in a coherent and policy-oriented strategy.

## What is ‘statistics’?

“Factual truth … is political by nature. Facts and opinions, though they must be kept apart, are not antagonistic to each other; they belong to the same realm. … Seen from the viewpoint of politics, truth has a despotic character. It is therefore hated by tyrants, who rightly fear the competition of a coercive force they cannot monopolize, and it enjoys a rather precarious status in the eyes of governments that rest on consent and abhor coercion. Facts are beyond agreement and consent, and all talk about them – all exchanges of opinion based on correct information – will contribute nothing to their establishment. Unwelcome opinion can be argued with, rejected, or compromised upon, but unwelcome facts possess an infuriating stubbornness that nothing can move except plain lies.” (Arendt, 1967, p. 7).

Statistics are by nature political in a special way; they are the object of opinion, but without themselves being influenced by opinions and interests. How can statistics do justice to this difficult task of being factual information in the sense of Hannah Arendt? How can facts be politically relevant but not politically driven?

The answer to these questions is multi-layered:

- It includes first and foremost good mathematical-statistical *methodology*
- and
- a modern *technology* based on reliable data sources and efficient processing procedures.
  But that is not all:
- Statistical processes require a sustainably maintained *infrastructure*, be it for research on methods and development of technologies, be it for the ‘fabrication’ of statistical information.
- Quality of statistical information proves itself in the ‘fitness for purpose’ test: Are the facts provided relevant, are they correctly understood, do they meet (realistic) expectations? This dimension of quality is achieved as a goal when all parties involved, i.e. the producers and the users of facts, data, trends, speak a common *language.*

**Fig. 1:**
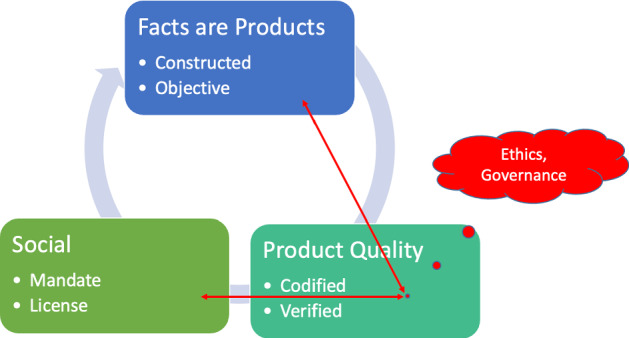
Parameters of excellent statistics

It is therefore necessary to approach the task with several objectives, which are partly in conflict to each other, and considered from different angles at the same time (see Fig. 1). If statistical results are not measurements conceived according to a naïve realism, but are crucially convention-dependent quantifications, how can they nonetheless be objective, neutral, and of trustworthy quality? How can the quality of statistical products defined in this way be achieved, guaranteed and communicated? What conditions must be met so that this theoretically possible quality is actually realised? Last but not least, which ethics, values and governance matter, is there a statistics-friendly or hostile ‘culture’?

## Statistics: old school or trendy?

Statistics is called upon to position and assert itself as an independent discipline in association with other data sciences. This is about cooperation, new possibilities and synergies. From a purely methodological-technical perspective, the differences between data science, artificial intelligence and statistics appear less serious. Rather, it is other factors such as values, quality definition or professional self-image that make the difference.^2^ Both on the side of the producers of quantitative information and on the side of the users, there are very different ‘data worlds’ in this respect, which becomes particularly clear in the comparison of the 'big data world' with official statistics (Radermacher 2021d).

## What is on the political agenda?

When analysing the role of data, statistics and decisions in a crisis (such as the Covid-19 pandemic), it is necessary to include policy programmes. This can be done, for example, with the help of the following recent strategically oriented policy plans:

- Coalition agreement of the German Federal Government^3^
- European Data Strategy,^4^ Europe’s Digital Decade^5^ of the European Commission

It is remarkable how absent ‘statistics’ is from these important plans, not only in terms of methods, but also in terms of infrastructure or language. Apparently, the dominant idea among those who develop and discuss such strategies is that nowadays data is ubiquitous (i.e. no longer needs to be collected), that intelligent algorithms automatically do the work of filtering the signals from the noise, and that it is a (meaningful) goal of digital education that every citizen has the skills of a data scientist and can produce his or her own statistics.

As a prerequisite for promoting improved data practices, there needs to be a deeper and broader analysis and understanding of what role data plays in contemporary society (Beaulieu and Leonelli 2021; Diaz-Bone and Horvath 2021; Henneguelle and Jatteau 2021). In this context, it is by no means sufficient to devote attention to the technical processes, the economics of data or similar. Likewise, it is not enough to focus the ethical and legal questions on the protection of individual privacy. With a proliferation of data, new dynamics are triggered that lead to the replacement of the previous well-ordered (infra)structures of statistical production based in the public sector by other forms of (decentralised and individual) use and production of data. The statistical logic of the past two centuries is being replaced by a data logic. Even if this statement comes across as too pointed, it nevertheless leads to an examination of the risks contained in this trend and measures to counter them effectively (Radermacher 2021b, 2021c).

## Dare (more) progress

In order to achieve progress, which is made possible with and through statistics, different initiatives are needed at the same time. Only a short list of recommendation points and activities can be made here. Nevertheless, this list should give an overview of their breadth and diversity:

### Methodology

- Strengthening cooperation between method development and application
- Promote links between data science and statistics
- Use complementarity between survey statistics and analysis of existing sources.

### Technology

- Advance and connect efficient technologies in all possible forms (especially AI, machine learning)
- Develop quality management for newer technologies
- Apply consequently an Open Data Strategy (access for research while protecting privacy of data).

### Infrastructure

Official statistics is a public infrastructure that (like rail transport) needs to be maintained, expanded and modernised. The following tasks and questions are up for solution:

- Balance decentralisation and centralisation tendencies in the implementation of digitalisation offensives
- Data Stewardship: make use of knowledge and capacities in existing institutions
- From official to public statistics: coordinate the larger system by a chief statistician^6^
- 'Data Institute': make use of knowledge and capacities in existing institutions and enhance networks between research and application
- Establish a forward looking programming of public statistics
- Governance: modernise the German Federal Statistical Law adapting to modern standards, such as the EU Statistics Regulation 223 (European Union, 2015).

### Language

- Invest in Data Literacy, support activities such as International Standard Development^7^ or Data Literacy Charters^8^
- Include statistical expertise in political expert panels.

### Ethics, values

- Promote the existing principles and guidelines for professional ethics^9^ and good governance^10^
- Review the guidelines and revise them as needed to cover newer methods in data science.

### Data culture

Overall, it is about actively promoting a data culture. This goal goes far beyond conveying ‘digital skills’. In fact, in many cases, corresponding activities have already been initiated and have produced concrete results. Such activities should be built upon and further networked and strengthened (Radermacher 2022).

## Improving the representation of scientific interests

All in all, the statistical community should become aware of and face the task of proactively offering its contribution to solving the upcoming critical transformation processes in the crises of the coming years. Statistics has a lot to offer in terms of potential solutions. Power brings with it responsibility.

Dealing with this will be the challenge of the statistical societies in the near future. Strategic guidelines such as a data manifesto^11^ could provide us with orientation.

## Footnotes

1 The book “Kunst Aufräumen” (Wehrli, 2002) may serve as an illustration.

2 See for example https://www.youtube.com/watch?v=uHGlCi9jOWY.

3 https://www.spd.de/koalitionsvertrag2021/.

4 https://ec.europa.eu/info/strategy/priorities-2019-2024/europe-fit-digital-age/european-data-strategy_en.

5 https://ec.europa.eu/info/strategy/priorities-2019-2024/europe-fit-digital-age/europes-digital-decade-digital-targets-2030_en.

6 See W. Radermacher Technical Discussion in https://download.statistik-berlin-brandenburg.de/0e2a6bd7d6fd44ce/b43aa17fd047/Zeitschrift-20210304.pdf, p. 38.

7 See https://sagroups.ieee.org/7015/.

8 See for example https://www.stifterverband.org/sites/default/files/data-literacy-charter.pdf.

9 https://www.isi-web.org/about/policies/professional-ethics.

10 https://unstats.un.org/unsd/dnss/gp/fundprinciples.aspx.

11 https://rss.org.uk/policy-campaigns/policy/our-data-manifesto/.
